# Modeling diameter at breast height of Chinese fir (*Cunninghamia lanceolata*) using UAV LiDAR data in Southern China

**DOI:** 10.3389/fpls.2025.1546055

**Published:** 2025-10-02

**Authors:** Ziyang Liu, Dongbo Xie, Zheyuan Wu, Linyan Feng, Xingyong Liao, Yongjun Wang, Wendong Zhu, Ram P. Sharma, Liyong Fu

**Affiliations:** ^1^ Research Institute of Forest Resource Information Techniques, Chinese Academy of Forestry, Beijing, China; ^2^ Chengdu Academy of Agriculture and Forestry Sciences, Chengdu, China; ^3^ Institute of Forestry, Tribhuvan University, Kathmandu, Nepal

**Keywords:** *Cunninghamia lanceolata*, airborne LiDAR, diameter at breast height modeling, growth stage, nonlinear mixed-effects model

## Abstract

Large-scale prediction of tree diameter at breast height (DBH) using airborne LiDAR remains constrained by models that inadequately address differences in tree growth stages and regional ecological variation. Existing approaches often overlook non-linear growth patterns and hierarchical spatial effects, thereby limiting predictive accuracy and scalability. In this study, we developed a DBH estimation model tailored for *Cunninghamia lanceolata* forests by integrating field-measured DBH with corresponding airborne LiDAR data collected from 26,768 trees across 130 plots in Guangdong Province, China. To capture growth-stage variability, a dummy variable approach was implemented to enable stage-specific adjustments within the model. Moreover, a two-level linear mixed-effects model was employed to account for nested spatial heterogeneity at both regional and stand levels. Competing model structures were rigorously evaluated using Akaike Information Criterion (AIC) and multiple error metrics, and the final model performance was validated with an independent dataset. Our results demonstrate that incorporating growth-stage differentiation and multilevel random effects significantly enhances model accuracy, with additional improvements observed upon including stand density and crown width indicators. The final model outperformed traditional approaches, effectively capturing spatial and ontogenetic variability. This study provides a methodological foundation for improving DBH estimation of *Cunninghamia lanceolata* using airborne LiDAR data. While further validation is needed, the modeling framework may also offer a potential basis for future applications using UAV-borne LiDAR platforms in similar forest environments.

## 1 Introduction

Forests play a crucial role in carbon sequestration and climate regulation, making accurate forest inventory data essential for sustainable forest management (Bonan, 2008). Diameter at breast height (DBH) is a key metric for forest management, providing essential information for estimating tree stock, biomass, and carbon storage (Fischer and Traub, 2019). Traditional DBH measurement using manual tools such as calipers is accurate but inefficient, making it unsuitable for large-scale forest resource assessments (Popescu and Wynne, 2004).

The advancement of remote sensing technologies has significantly improved forest inventory efficiency and scalability (Gonzalez-Benecke et al., 2014). LiDAR excels at capturing forest structure, providing valuable insights into forest metrics and stand characteristics (Faridhouseini et al., 2011). Previous studies have demonstrated the utility of LiDAR in estimating stand-level metrics (Falkowski et al., 2006; González-Ferreiro et al., 2012) and individual tree attributes (Aubry-Kientz et al., 2019; Wang et al., 2020; Yuwei et al., 2021). However, airborne LiDAR cannot directly measure DBH (Liu et al., 2018), and while handheld or backpack LiDAR improves efficiency, it is limited by understory complexity and small-scale applicability (Bu and Wang, 2016; Hui et al., 2024). Furthermore, existing DBH models primarily focus on stand-level metrics and often fail to capture individual tree-level variability, particularly across different growth stages and environmental conditions (Piao et al., 2018; Zhang et al., 2023). This underscores the need for more refined models that can integrate multiple influencing factors, including site-specific differences and within-stand competition, to improve predictive performance and generalizability.

DBH can be categorized into stand-level and individual tree-level metrics, with individual tree DBH being critical for detailed forest management and improved precision in forest operations (Sparks and Smith, 2022). While tree height and crown width have been widely used as DBH model predictors (Filipescu et al., 2012; Sharma et al., 2019; Nigul et al., 2021; Iizuka et al., 2022; Tinkham et al., 2022; Lele et al., 2023), Growth rates differ across age classes (Mu et al., 2017; Liu et al., 2020a), and models incorporating growth stage effects tend to improve predictive accuracy (Mu et al., 2017; Xiao et al., 2022). Similarly, competition within stands alters growth strategies, with intense competition favoring height over diameter growth (Kunstler et al., 2011; Wertz et al., 2020). addition to growth stage and competition, regional differences such as climate, soil type, and topography also significantly affect tree growth patterns (Canham et al., 2018; Luo et al., 2024). Therefore, developing a modeling framework that incorporates multi-scale variability and site-specific influences is essential for improving DBH estimation at both the stand and individual tree levels.

Chinese fir is a commercially and ecologically important species native to southern China. Accurate large-scale DBH estimation for Chinese fir can improve forest resource management and ecological monitoring. This study aims to: (1) develop a two-level nonlinear mixed-effects model, using blocks and plots as random effects, to enhance the accuracy of individual tree DBH estimation for Chinese fir. improve individual tree DBH estimation accuracy; (2) evaluate the influence of growth stage and competition intensity on DBH estimation; and (3) propose a scalable DBH estimation method using airborne LiDAR data to support large-scale forest management and resource assessment.

## 2 Materials and methods

### 2.1 Data description

**Figure 1:**
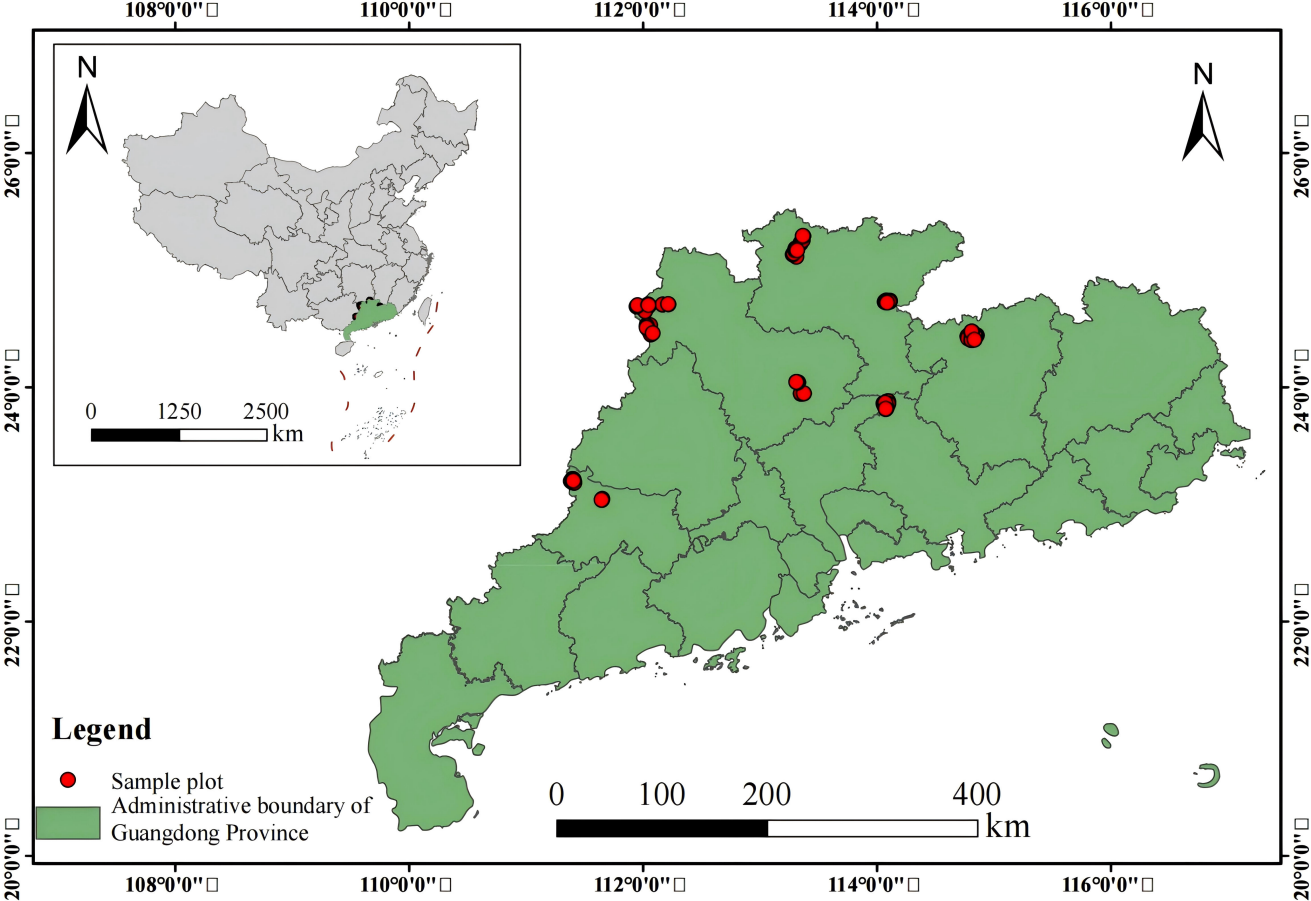
Plot of site location.

The study area is located in five forested regions in Guangdong Province, China (20°09′~25°31′N and 109°45′~117°20′E) (**Figure 1**). The study area is predominantly situated in the northwestern part of Guangdong, characterized by a landscape of mountains and low hills. Slopes within the region range from 10° to 40°, with generally high relief in the north and lower relief in the south. Precipitation in this region is concentrated from April to September, with an average annual rainfall of 1,777 mm. The highest recorded average annual rainfall can reach 2,321 mm. The region has an average annual temperature of 21.8°C, with warm temperatures throughout the year and abundant rainfall. The soil in the study area is primarily red and yellow loam. This area is an important part of the middle subtropical zone in China and one of the key distribution areas for Chinese fir.

In March 2024, we selected representative Chinese fir(*Cunninghamia lanceolata)* plantation forests and established 130 sample plots containing a total of 26,768 trees, each covering an area of 666.67 m^2^. The basic site conditions of the sample plots were recorded, and for each tree within the plots (with a DBH > 5 cm), we measured the tree height(H), diameter at breast hight(DBH), height crown base, and crown width(CW) in four perpendicular directions. The exact location of each tree within the plot was also recorded.

In June 2024, we collected data from all the plots using airborne LiDAR. The equipment used to collect the data was the AS-1300HL LiDAR system, which is equipped with the Rigel VUX-1LR laser scanner. This system operates at a wavelength of 1550 nm, with a pulse duration of 3.5 ns and a laser beam divergence of 0.5 m rad. The pulse repetition frequency is set at 50 kHz, the maximum scan angle is 30°, and the scanning frequency is 49 Hz. A grid-pattern flight path was used, with a 50% side overlap of the point clouds. The average flight speed was 10 m/s, and the average point cloud density was 200 points per square meter.

The independent validation method is one of the most commonly used methods to test the generalization ability and fitting effectiveness of a model, we used 70% of the data as modeling data and 30% as independent validation dataset, Collected UAV LiDAR data were preprocessed using LiDAR 360 software. Noise filtering of the point cloud was performed using a neighborhood-based approach with a threshold set at three times the standard deviation. Ground points were identified using an improved progressive triangulated irregular network (TIN) densification filtering algorithm, enabling point cloud normalization. Individual tree segmentation was conducted using a distance-based single tree segmentation algorithm.

**Figure 2:**
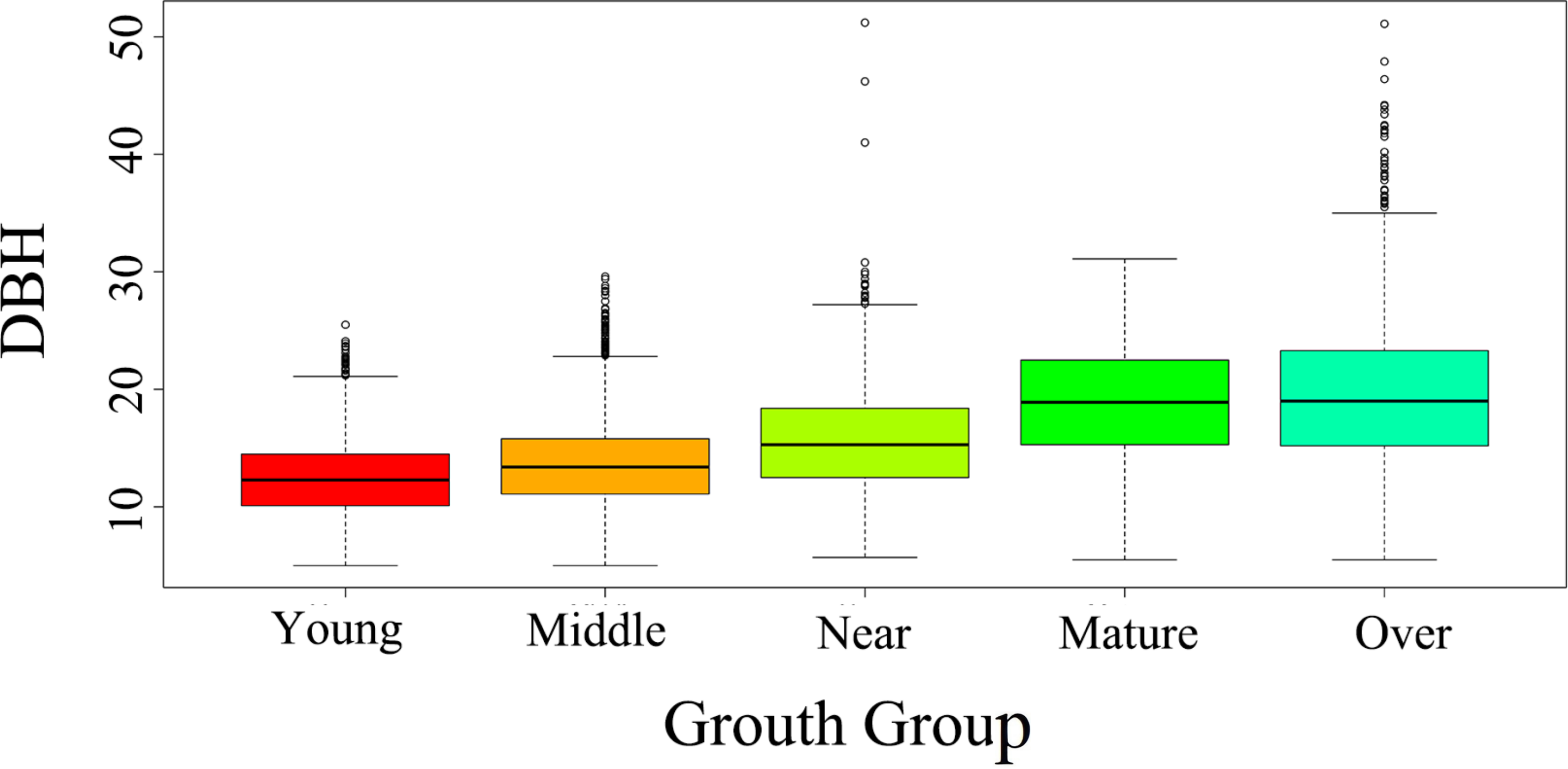
The box plots of the distribution of breast diameter with age groups.

In cases of high stand density, where the point clouds might become densely packed, we performed an additional manual segmentation step to ensure accurate delineation of individual trees. This secondary segmentation ensured that overlapping or closely spaced trees were properly separated, maintaining the quality and accuracy of the segmentation process. The correlation between the LiDAR-derived tree height (H) and the field-measured values reached 0.79, and the overall correlation coefficient between canopy width and measured values was 0.63. Stand density indicators were the number of plants per hectare based on sample plot surveys, and the growth stage of fir trees was determined by the age group. A detailed summary of the data is given in **Table 1**. Box plots of the distribution of breast diameter with age groups are shown in **Figure 2**.

**Table 1:** Summary statistics of data acquired by both methods (UAV LiDAR and ground measurement).

	Training data					Testing data				
	DBH	LH	LCD	S	A	DBH	LH	LCD	S	A
Max.	51.20	32.58	12.29	5311	40	44.10	29.76	16.47	5311	40
Mean	14.69	13.14	2.32	2878	18.24	14.71	13.13	2.33	2866	18.28
Min.	5.00	3.82	0.04	433	6	5.00	3.51	0.046	433	6
Stand error	5.02	3.90	1.40	916	9.82	5.01	3.91	1.42	919	9.70

DBH, stem outside bark diameter at 1.3 m height (cm); LH, tree height by lidar (m); LCD, Crown width by lidar (m); S, number of living trees per hectare (ha); A: the age of an individual tree (years).

### 2.2 Modeling Methods

#### 2.2.1 Basic models

Tree H and DBH are highly correlated (Hao et al., 2016), and airborne LiDAR excels at providing accurate height data. To model the relationship between DBH and LiDAR-derived height, five candidate models were selected to describe the curvilinear relationship between DBH and H: linear, Weibull, logistic, Wykoff, and Gompertz models (Xue-hua et al., 2013).These models have been widely applied in forestry for capturing tree growth patterns and height-diameter relationships. Since DBH is also frequently used to estimate tree height, we reversed the equations of several established H-DBH models (Filipescu et al., 2012) to generate additional candidate models for DBH estimation. Ultimately, a total of ten candidate base models were identified. The mathematical expressions of these models are presented in **Table 2**.

**Table 2:** Basic DBH - height models evaluated.

Model	Equation	Name
Two-parameter models		
M1	D=a+bH	Linear
M2	$D=aH^{b}$	Allometric growth model
M3	$D=a{\text{e}}^{bH}$	Exponential functions
M4	D=alnH+b	Logarithmic functions
M5	$D=e^{b\ln((H-1.3)/a)}$	Tuan et al., 2019
M6	$D=\frac{b}{\ln H-a}-1$	Wykoff et al, 1982
Three-parameter models		
M7	$D=a+bH^{c}$	Allometric model with intercepts
M8	$D={\left[\begin{array}{c} \ln\left(\frac{\frac{H}{a}-1}{\frac{1}{\text{b}}}\right) \end{array}\right]}^{\frac{1}{c}}$	Yang et al., 1978
M9	$D=\frac{\ln\left[1-{\left(\frac{H}{a}\right)}^{\frac{1}{c}}\right]}{-b}$	Clarke and Haines, 1995
M10	$D=\frac{b}{\ln(H-1.3)-a}-c$	Exponential

*D*, diameter at breast height; H, tree height; *a, b*, and *c* are parameters to be estimated.

#### 2.2.2 Variable selection

LiDAR-derived metrics were classified into two primary categories: height-related metrics (e.g., tree height) and canopy-related metrics (e.g., crown width, canopy area, and canopy volume). These metrics are widely used in forestry due to their strong ecological interpretation and demonstrated correlations with DBH (Brolly et al., 2012). To account for competition effects, stand density was extracted by counting individual trees identified through LiDAR point cloud segmentation within each sample plot. Studies have shown that LiDAR-derived metrics often exhibit multicollinearity (Silva et al., 2016; Stitt et al., 2022). To mitigate this issue, we first conducted a Variance Inflation Factor (VIF) test and retained only those variables with VIF< 5 (Peereman et al., 2021). Subsequently, a Pearson correlation analysis was performed on the selected variables to identify the optimal set for model development. Once the base model (**Table 2**) was established, additional variables were gradually introduced through the reparameterization or other statistical methods to optimize the models’ evaluation metrics (Equations 3-6).

#### 2.2.3 Dummy variable modeling

Dummy variables allow for the inclusion of categorical variables into the model, i.e., growth stages or age groups can be included in the form of dummy variables to influence the model. Tree growth patterns can vary across different growth stages, as observed in species, such as *Picea abies* (Konopka et al., 1987)*, Calocedrus formosana* (Chiu et al., 2015), and *Larix* (Orzeł, 2007). Chinese fir is a typical fast-growing species, and according to age, we categorized fir into five growth stages: young (1–10 years), middle-aged (11–20 years), near mature (21–25 years), mature (26–36 years), and overmature (>36 years), and we used this to characterize the effect of growth stage on the model of DBH. Therefore the DBH model was developed by incorporating the tree growth stage as a dummy variable. The dummy variable model is formulated as shown in Equation 1.


(1) $$DBH=\sum_{i=1}^{n}(G_{i}a_{i})g(LH,C,c_{i}))+\varepsilon$$

Where:*a_i_* are model parameters and *G_i_* is the dummy variable which can be 0 or 1. *i* =1,2,3,4,5. When *G_1_ = 1,G_2_,G_3_,G_4_* and *G_5_* are 0, and so on, *G_1_* refers to young fir forests and *G_5_* refers to over-mature fir forests, *g(LH, C, c_i_)* represents a DBH model with *LH* and *C* as variables and *c_i_* as parameter, *HL* and *C* are independent variables in the model, and *ε* represents the error term.

The established dummy variable model not only incorporates the individual tree variables and competition variables but also accounts for the impact on the changes in DBH. The optimal form of the model and its fitting performance are determined and selected based on model evaluation metrics (Equations 3-6).

#### 2.2.4 Mixed-effects modeling

A combination of factors, such as regional climatic conditions, significantly influences DBH growth (Liu et al., 2020b). Even within the same region, subtle environmental and competitive differences between sample sites can lead to variations in DBH growth. Therefore, it is essential to consider both inter-regional and intra-regional differences when modeling DBH growth. To better account for these hierarchical influences, we implemented a two-level nonlinear mixed-effects model that captures both broad regional variability and localized site-specific effects. This approach enhances the accuracy of DBH growth estimation by incorporating nested random effects, providing a more refined representation of the underlying biological and environmental processes. In this model, tree growth is expressed as a function of both broad regional influences and localized site-specific factors. By integrating random effects at both the regional and sample plot levels, The general form of the two-level nonlinear mixed-effects model is presented in Equation 2.


(2) $$\left\{ \begin{array}{l} DBH_{ijk}=f_{ilk}(D_{1}(LH_{ijk},C_{ijk},\delta_{ijk}),u_{ijk},v_{ijk})+\varepsilon_{ijk}\\ u_{i}=(u_{1i},u_{2i},u_{3i})'∼N(0,\psi_{1}),\\ v_{i\text{j}}=(u_{1ij},u_{2ij},u_{3ij})'∼N(0,\psi_{2}),\\ \varepsilon_{ijk}∼N(0,\sigma^{2}),\\ i=1,\ldots ,9,j=1,\ldots 130. \end{array}\right.$$

where DBH_ijk_ represents the diameter at breast height (DBH) of the *k-th* tree in the *j-th* sample plot of the *i-th* region.; *f_ijk_* is DBH model that includes two-level random effects; $D_{1}(LH_{ijk},C_{ijk},\delta_{ijk})$ is the model with *LH_ijk_* and *C_ijk_* as variables and with $\delta_{ijk}$ as the parameter to be estimated. *u_i_* and *v_ij_* are random effects vectors indicating the level of sample plots in the region and within the region, respectively. $\varepsilon_{ijk}$ denotes the random error in *k-th* tree diameter at breast height for *j-th* sample plots in *i-th* region, $\psi_{1}$ and $\psi_{2}$ refer to the corresponding random-effects variance-covariance matrixes, and $\sigma^{2}$ refers to the random error variance. The random effects assumption and the error term assumption are independent of each other and each follows a normal distribution.

Mixed-effects model was fitted by the nlme package in R 4.2.3. Model parameters were estimated using the restricted maximum likelihood (REML) method (Corbeil and Searle, 1976), which provides unbiased estimates of variance components by accounting for the degrees of freedom consumed in estimating fixed effects. After fitting the model, we used the *AIC* and the likelihood ratio test(*LRT*) to evaluate model performance and select the best-fitting model (Fang and Bailey, 2001). *AIC* was used to compare model fit by balancing model complexity and goodness of fit, while *LRT* assessed the significance of additional model terms.

### 2.3 Model evaluation

In this study, we used an independent validation approach to model validation, and we randomly divided the data into two datasets, with 70% of the data used for model fitting (18738 observations) and the other 30% for model validation (8030 observations). The model evaluation metrics were *AIC* (Akaike Information Criterion), *R^2^* (Coefficient of Determination), *RMSE* (Root Mean Squared Error) and *TRE* (Total Relative Error).


(3) AIC=−2lnl+2p

(4) $$R^{2}=1-\frac{\sum_{i=1}^{n}{\left(D_{i}-\hat{D_{i}}\right)}^{2}}{\sum_{i=1}^{n}{\left(D_{i}-\bar{D}\right)}^{2}}\;(\frac{n-1}{n-p})$$

(5) $$RMSE=\sqrt{\frac{\sum_{i=1}^{n}{\left(D_{i}-{\hat{D}}_{i}\right)}^{2}}{n-p}}$$

(6) $$TRE=\frac{\sum_{i=1}^{n}{\left(D_{i}-\hat{D_{i}}\right)}^{2}}{\sum_{i=1}^{n}D_{i}^{2}}$$

where l is the maximum likelihood of the model; n is the number of observations;*p* is the number of parameters in the model;*D_i_* is the *i*-th observed value of the DBH; ${\hat{D}}_{i}$ is the *i*-th predicted value of the DBH; $\bar{D}$ is the mean value of the DBH.

## 3 Results

### 3.1 Selected variables and their correlations with DBH

**Figure 3:**
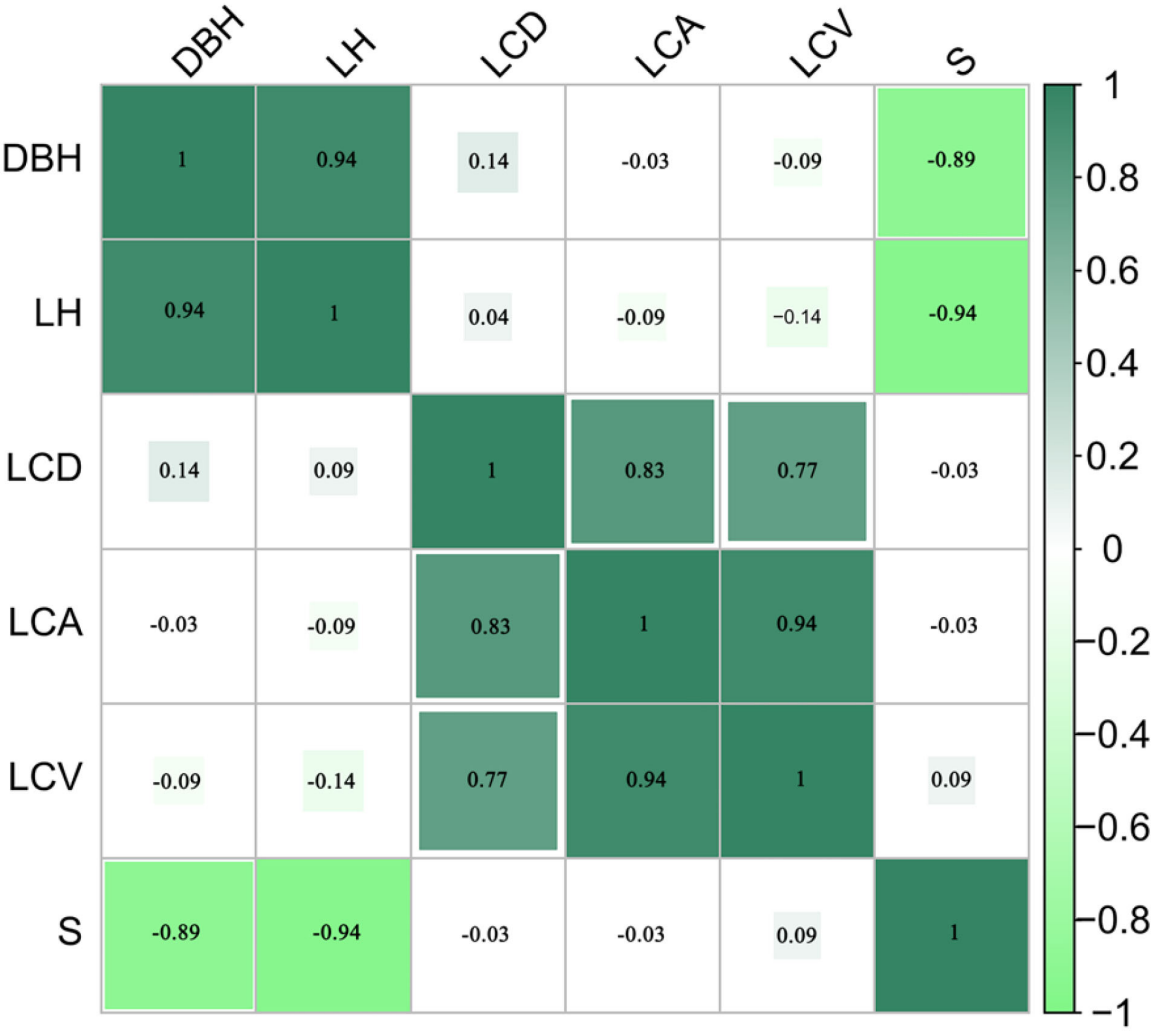
The correlation heatmap between diameter at breast height and various variables is provided. DBH represents the ground truth diameter at breast height (DBH), LH denotes tree height, and LCD, LCA, and LCV represent canopy width derived from LiDAR data. S refers to stand density.

After VIF testing, the final retained variables were CW and S. The results of the VIF values are shown in **Table 3**. Pearson correlation analysis revealed that LiDAR-derived tree height exhibited the highest correlation with ground-measured DBH, indicating its strong predictive power. Both stand density and CW also showed correlations with DBH, consistent with their biological roles in tree growth — CW representing the proportion of stand growth and stand density reflecting competition intensity within the stand. In contrast, canopy area and canopy volume exhibited weaker correlations with DBH, suggesting that they contribute less to explaining diameter variability. To enhance model generalizability and reduce overfitting, we ultimately selected tree height, canopy width, and stand density as covariates in the DBH model. The correlation heatmap between DBH and the various variables is shown in **Figure 3**.

**Table 3:** VIF values for each variable.

Variable	LH	LCD	S
VIF	1.799	1.200	1.600

### 3.2 Generalized OLS model

As shown in **Table 4**, Model M7 was selected as the optimal base model due to its highest R² and lowest RMSE and AIC values, indicating that the most appropriate relationship between individual tree DBH and tree height follows an intercept plus power function. To account for the effects of multiple variables on DBH estimation, variables were gradually added to M7 using a stepwise reparameterization procedure based on the continuous product of power functions. A total of ten reconstructed model forms were compared, and the extended version of the base model, known as a generalized OLS model (Equation 7), demonstrated superior predictive performance over the best base model (M7) (**Table 5**). An F-test comparing Equation 7 and M7 indicated that the generalized model improved predictive ability and statistical significance (F = 470, p< 0.001). Although the improvement in predictive accuracy was modest, the new model enhanced interpretability and increased robustness under varying site conditions.

**Table 4:** Fit indicators of the base models.

Model	Training data		Testing data		*AIC*
	*R^2^*	*RMSE(cm)*	*R^2^*	*RMSE(cm)*	
M1	0.6028	3.160	0.6027	3.1535	69655
M2	0.6024	3.165	0.6023	3.1589	69701
M3	0.5989	3.1790	0.5971	3.1797	69821
M4	0.4131	3.8456	0.4143	3.8337	74979
M5	0.6041	3.1730	0.6004	3.1667	69770
M6	0.2265	9.5978	0.2101	9.6921	99772
M7	0.6053	3.1530	0.6049	3.1489	69604
M8	0.5916	3.2078	0.5919	3.1999	70066
M9	0.5635	3.3163	0.5624	3.3137	70967
M10	0.5635	3.3163	0.5624	3.3137	70967

**Table 5:** Fit indicators of base model (M7) and Equation 7.

Dataset	Indicators	M7	Equation 7
Train set	*RMSE (cm)*	3.153	3.049
	*TRE (%)*	4.302	4.012
	*R^2^*	0.6053	0.6301
Test set	*RMSE (cm)*	3.151	3.059
	*TRE (%)*	4.290	4.051
	*R^2^*	0.6052	0.6261
Evaluation	*AIC*	69604	68698
*F* (M7 VS Equation 7)	470		
*p-value*	<0.001***		

The expression for this model is as follows:


(7) $$DBH=2.520+3.842LH^{0.894}LCD^{0.062}S^{-0.152}$$

### 3.3 Dummy variable model

Dummy variables were introduced to represent different age groups, allowing the model to account for growth stage-specific variations. To avoid overfitting and excessive model complexity, we incorporated dummy variables for only one parameter to balance model performance and simplicity. Through a systematic evaluation procedure, the model that applied dummy variables to parameter b demonstrated the best fit, as indicated by the lowest AIC. Parameter b reflects the growth rate adjustment across different age groups, suggesting that growth stage variations primarily influence the scaling factor rather than the base growth function. The model specification is as follows:


(8) $$\begin{array}{c} DBH=1.592+(3.901G_{1}+3.946G_{2}+4.052G_{3}+4.092G_{4}\\ +4.177G_{5})LH^{0.8567}LCD^{0.0539}G^{-0.1356} \end{array}$$

where: *G_1_, G_2_, G_3_, G_4_* and *G_5_* are dummy variables representing the different age classes of Chinese fir: young forest, middle-aged forest, near-mature forest, mature forest, and over-mature forest, respectively.

### 3.4 Mixed-effects model

**Figure 4:**
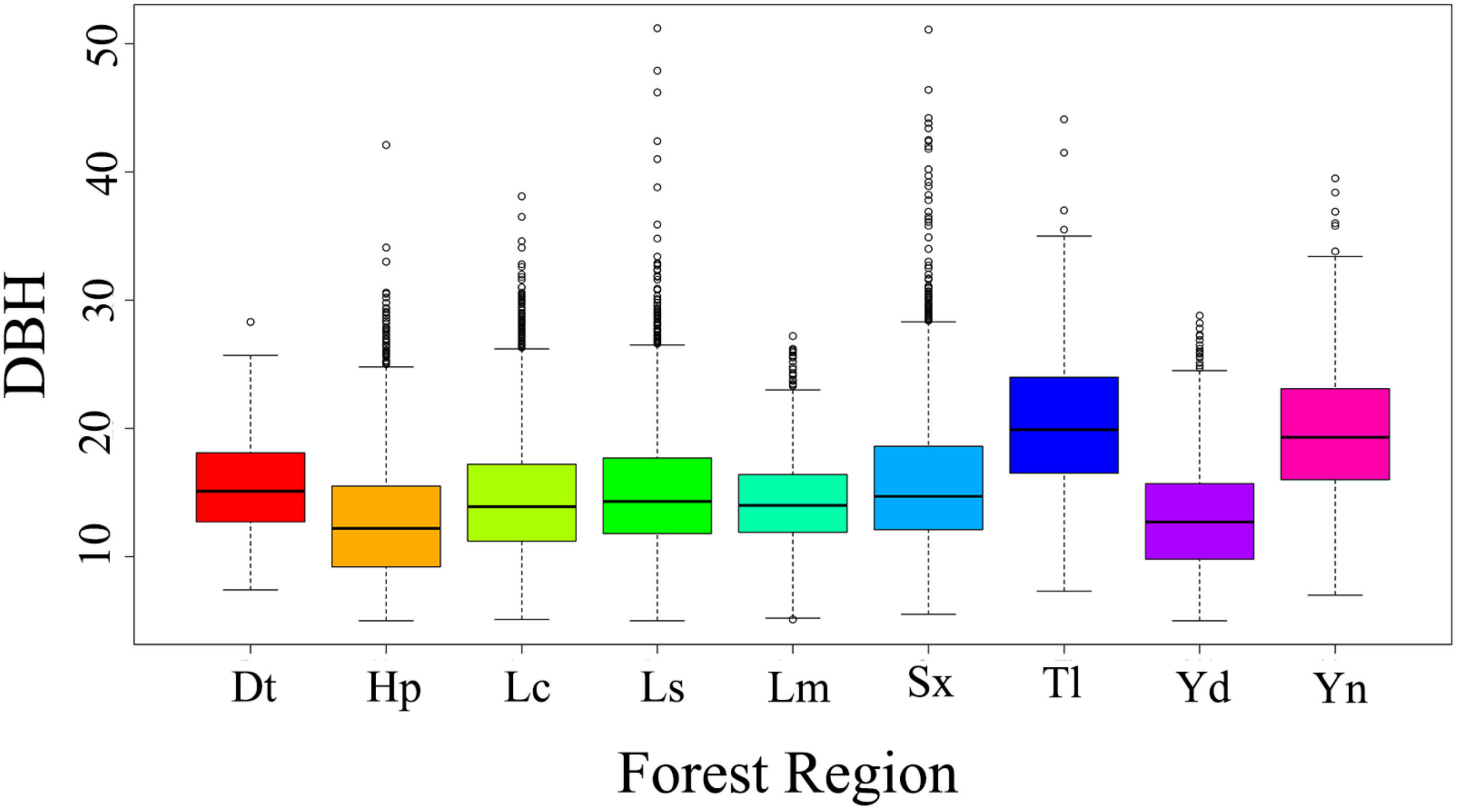
Distribution of DBH across different forest regions. Abbreviations represent the corresponding locations: Dt, Datang Industrial Zone; Hp, Heping County; Lc, Lechang County; Ls, Lianshan County; Lm, Longmen County; Sx, Shixing County; Td, Tongle Forest Farm; Yd, Yingde Forest Farm; and Yn, Yunan Forest Farm.

**Figure 4** presents the distribution of DBH across different forest regions. It is evident that DBH varies significantly among regions, with some regions (e.g., Tl, Sx) exhibiting greater DBH variability and higher median values compared to others (e.g., Hp, Dt). These differences highlight the necessity of incorporating regional random effects in the model. Based on Equation 8, we developed a two-level random effects model incorporating region-level and plot-level variability. Various model structures were tested and evaluated based on optimal model selection criteria. model achieved the best performance when regional random effects were applied to all parameters, while plot-level random effects were applied only to parameter a. This model had the highest log-likelihood (-33,466) and the lowest AIC (66973). To further quantify the contribution of random effects and assess the impact of incorporating mixed effects, we conducted a LRT. Compared to the baseline model, introducing regional random effects significantly improved model performance (LRT = 1600, p< 0.001), confirming the importance of capturing broad-scale site variation. Furthermore, when comparing the single-level regional random effects model to the two-level random effects model, the model was further optimized (LRT = 404, p< 0.001), confirming the effectiveness of the two-level structure. The evaluation indicators of the model are shown in **Table 6**. Therefore, the two-level mixed-effects model of the DBH expression is:

**Table 6:** Fit indicators of Equations 8, 9.

Dataset	Indicators	Equation 8	Equation 9
Train set	*RMSE (cm)*	3.032	2.794
	*TRE (%)*	3.965	3.348
	*R^2^*	0.6651	0.7002
Test set	*RMSE (cm)*	3.048	2.822
	*TRE (%)*	4.002	3.408
	*R^2^*	0.6497	0.7025
Evaluation	*AIC*	68553	66973
	*Loglik*	-34266	-33466


(9) $$\begin{array}{l} DBH_{ijk}=4.462+u_{ik1}+v_{ijk}+(0.1906G_{1}+u_{ik2}+0.2008G_{2}+u_{ik3}\\ +0.2035G_{3}+u_{ik4}+0.2138G_{4}+u_{ik5}+0.2307G_{5}+u_{ik6})\\ LH^{\left(1.461+u_{ik7}\right)}LCD^{\left(0.0429+u_{ik8}\right)}S^{\left(0.0072+u_{ik9}\right)} \end{array}$$

where 


$$u_{ik}=\left[\begin{array}{c} u_{ik1}\\ u_{ik2}\\ u_{ik3}\\ u_{ik4}\\ u_{ik5}\\ u_{ik6}\\ u_{ik7}\\ u_{ik8}\\ u_{ik9} \end{array}\right]\sim N\left\{\left[\begin{array}{c} \begin{array}{c} 0\\ 0\\ 0\\ 0 \end{array}\\ \begin{array}{l} 0\\ 0\\ 0\\ 0\\ 0 \end{array} \end{array}\right],{\hat{\Psi }}_{1}=\left(\begin{array}{ccccccccc} \begin{array}{r} 5.7316 \end{array} & -8\mathrm{.7969}\times 10^{-12} & -3.3661\times 10^{-10} & -1.9716\times 10^{-6} & -0.0166 & -3.7131\times 10^{-13} & 0.0001 & 7.4378\times 10^{-6} & 1.4204\times 10^{-7}\\ -8.7970\times 10^{-12} & 1.9669\times 10^{-22} & 7.2328\times 10^{-21} & 4.1066\times 10^{-17} & 3.4126\times 10^{-13} & 7.4771\times 10^{-24} & -1.0297\times 10^{-15} & -3.0543\times 10^{-15} & -6.2927\times 10^{-19}\\ -3.3661\times 10^{-10} & 7.2328\times 10^{-21} & 2.6918\times 10^{-19} & 1.5192\times 10^{-15} & 1.2651\times 10^{-11} & 2.7745\times 10^{-22} & -3.9401\times 10^{-14} & -1.1025\times 10^{-11} & -2.4434\times 10^{-17}\\ -1.9716\times 10^{-6} & 4.1066\times 10^{-17} & 1.5192\times 10^{-15} & 8.7126\times 10^{-12} & 7.1971\times 10^{-8} & 1.5769\times 10^{-18} & -2.3126\times 10^{-10} & -6.0891\times 10^{-8} & -1.4010\times 10^{-13}\\ -0.0166 & 3.4126\times 10^{-13} & 1.2651\times 10^{-11} & 7.1971\times 10^{-8} & 0.0006 & 1.3171\times 10^{-14} & -1.9593\times 10^{-6} & -0.0005 & -1.1592\times 10^{-9}\\ -3.7131\times 10^{-13} & 7.4771\times 10^{-24} & 2.7745\times 10^{-22} & 1.5769\times 10^{-18} & 1.3171\times 10^{-14} & 2.9001\times 10^{-25} & -4.3734\times 10^{-17} & -1.0801\times 10^{-14} & -2.616\times 10^{-20}\\ 0.0001 & -1.0297\times 10^{-15} & -3.9401\times 10^{-14} & -2.3126\times 10^{-10} & -1.9593\times 10^{-6} & -4.3734\times 10^{-17} & 1.312\times 10^{-8} & 2.491\times 10^{-8} & 5.5216\times 10^{-12}\\ 7.4378\times 10^{-6} & -3.0543\times 10^{-13} & -1.10252\times 10^{-11} & -6.0891\times 10^{-8} & -0.0005 & -1.0808\times 10^{-14} & 2.491\times 10^{-8} & 0.001 & -1.0368\times 10^{-10}\\ 1.4204\times 10^{-7} & -6.2927\times 10^{-19} & -2.4434\times 10^{-17} & -1.401\times 10^{-13} & -1.1592\times 10^{-9} & -2.616\times 10^{-20} & -5.5216\times 10^{-12} & -1.0369\times 10^{-10} & 1.3751\times 10^{-13} \end{array}\right)\right\}$$

$v_{ik}=v_{ik1}∼N(0,1.9409)$ $\xi_{ijk}∼N\left(0,7.8822G_{i}^{0.5}\Gamma_{i}G_{i}^{0.5}\right)$ where *DBH_ijk_* refers to the diameter at breast height of the *k-th* tree in the *j-th* sample plot in the *i-th* region. *G_1_~G_5_* denote dummy variables for the five growth stages of fir from juvenile to overstory. *LH, LCD*, and *S* denote the H of an individual tree corresponding to the diameter at breast height, the crown width, and the density of the stand in the sample plot, respectively.

**Figure 5:**
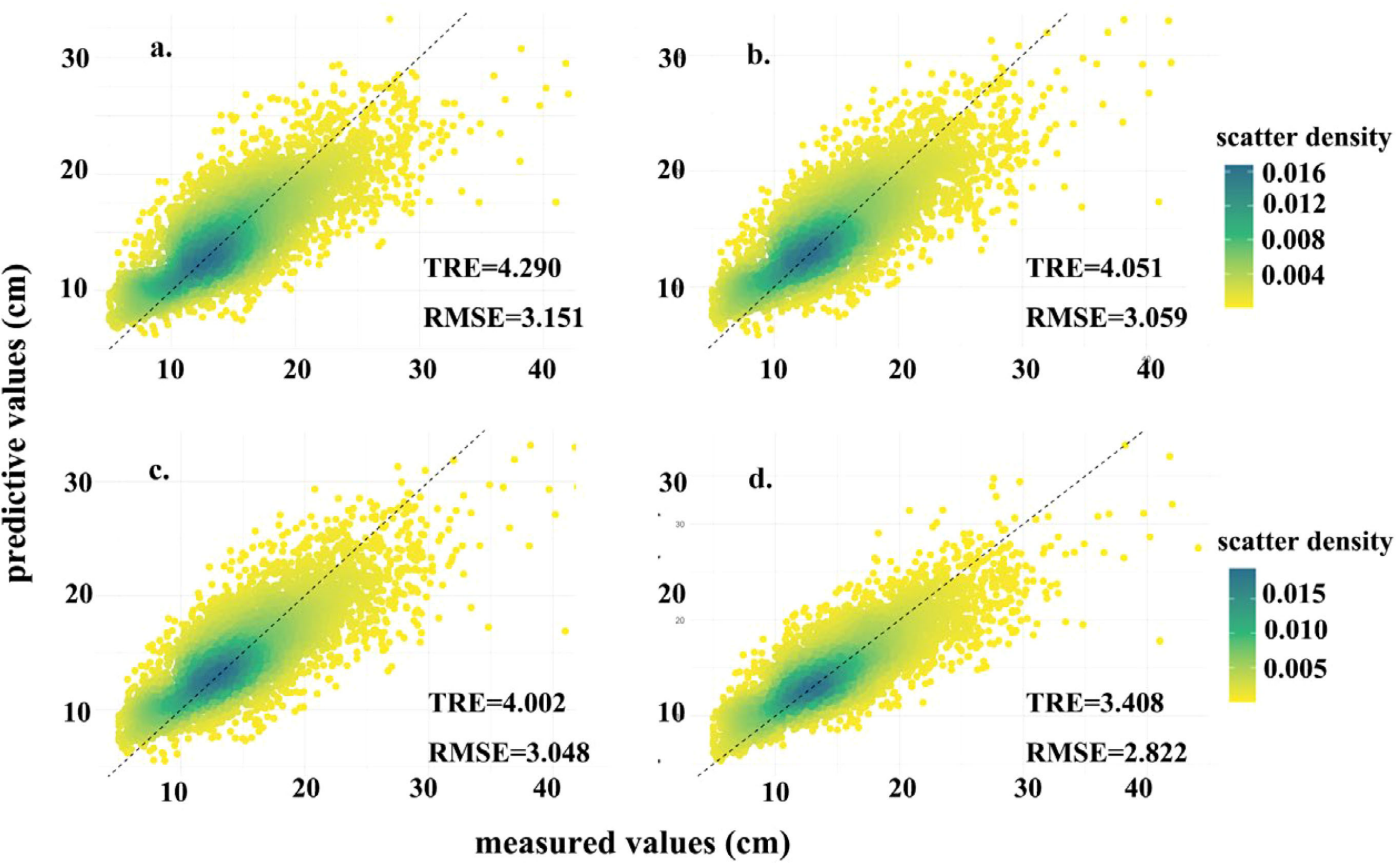
Predictive map **(a–d)** for M7, Equations 7–9 respectively, the reference line with a diagonal line of y=x.

According **Figure 5**, addition of the relevant variables improved the fit of the DBH model and reduced the error index, The model was gradually optimized by incorporating growth stages, regional effects, and plot-level variability. the final model R2 improving by 16.04% compared to the base model, indicating a substantial improvement in model accuracy and explanatory power. Furthermore, as the model was refined, the alignment between the predicted and observed values in the point plot improved noticeably. The points increasingly converged along the reference line x=y, suggesting that the model’s predictive performance became more accurate and reliable as additional hierarchical effects were incorporated.

## 4 Discussion

While traditional methods have provided valuable insights into DBH estimation, the need for large-scale, efficient assessment methods remains unmet (Liu et al., 2021). Existing LiDAR-based methods have shown promise in large-scale monitoring but face limitations in capturing individual tree metrics accurately, particularly under varying site conditions. To overcome these challenges, this study developed a general model for estimating the DBH of individual trees using terrestrial and airborne LiDAR data, enabling the rapid acquisition of a large number of Chinese fir DBH measurements through airborne LiDAR. The proposed model integrates allometric growth relationships and incorporates the effects of growth stage and regional variations, demonstrating both strong interpretability and high predictive accuracy. With the continuous advancement of smart forestry, this model provides reliable technical support for the rapid measurement of Chinese fir DBH.

UAV remote sensing offers high efficiency and large-scale coverage (Yan, 2020), but existing LiDAR-based methods still face limitations in individual tree-level DBH estimation (Lo and Lin, 2013). Previous studies on DBH estimation using LiDAR have focused primarily on stand-level mean DBH (Muhamad-Afizzul et al., 2019; Ozkan et al., 2022; Zhang et al., 2023).The challenge lies in the unstable relationship between LiDAR-derived metrics and individual tree DBH, making it difficult to identify consistent patterns and predictive variables. Interestingly, this study found a weak correlation between LiDAR-derived canopy width and DBH, which contrasts with the findings of some researchers (Kalliovirta and Tokola, 2005; Raptis et al., 2018), This discrepancy may stem from LiDAR’s superior ability to capture vertical forest structure while being less effective at representing horizontal structure (Bouvier et al., 2015; Coomes et al., 2017; Moran et al., 2018).

Tree growth strategies are influenced by both intrinsic factors (e.g., growth stage) and extrinsic factors (e.g., competition), which jointly determine resource allocation and growth efficiency (Vanninen, 2004). For a long time, the allometric growth relationships of trees have been a key focus in forest management and afforestation research, as these relationships are shaped by a complex interaction between genetic potential and environmental pressures (Matsushita et al., 2015; Sharma et al., 2019). Liu et al. (2020a)demonstrated that including age as a variable significantly improved DBH model performance for Larix species. Similarly, this study introduced growth stage as a dummy variable, yielding comparable improvements in predictive performance. However, the improvement was relatively modest, likely due to species differences or variations in model variables (Cao et al., 2024; Taye and Kelil, 2024). Competition also plays a critical role in tree growth strategies. Under high stand density, trees tend to prioritize height growth over radial growth, leading to suppressed DBH under competitive conditions (Boyden et al., 2009; Zhang et al., 2024). Incorporating plot density as a competition-related variable improved model fitting performance, highlighting the importance of accounting for stand competition in DBH models.

The two-level random effects introduced in this study include block-level and plot-level variations. The block-level effect accounts for broad-scale regional differences caused by factors such as climate, topography, and soil type, which influence tree growth patterns at a macro scale (Canham et al., 2018; Wang and Ibáñez, 2022; Luo et al., 2024). In contrast, the plot-level effect captures fine-scale environmental heterogeneity within blocks, such as microclimate and local competition intensity (Prior and Bowman, 2014; Latifi et al., 2015). To evaluate the contribution of the two-level effects, we compared the model’s performance with and without these random effects. When excluding the two-level effects, the model exhibited a substantial increase in the AIC the model exhibited a substantial increase in the AIC by 1580 and a reduction in the R² by 0.0528, indicating poorer model fit and predictive accuracy. (**Table 6**). This suggests that ignoring regional and plot-level variations results in increased model bias and reduced generalization ability. For instance, similar findings have been reported in other forest growth models. Fortin et al. (2016)demonstrated that accounting for hierarchical site variability improved the accuracy of tree growth predictions, particularly under varying environmental conditions. Therefore, the inclusion of two-level effects in this study reflects an essential methodological improvement that enhances both the interpretability and predictive power of the DBH model. Lo and Lin (2013) developed a DBH model incorporating competition and regional differences, showing strong interpretability, can also attest to this. Their model achieved higher overall accuracy, which may be attributed to differences in predictor variables and sample size. Lo et al. introduced a novel competition-related variable (LCI) to account for neighboring competitors, whereas our study incorporated plot density to reflect competition intensity. Additionally, our model encompassed all growth stages of Chinese fir, whereas Lo et al.’s model focused solely on mature and overmature stands.

Interestingly, the explanatory power of the density variable weakened after introducing random effects into the model (**Tables 7**, **8**). This may be due to the fixed effect of stand density being partially absorbed by block-level random effects, or it may reflect environmental variations in the influence of stand density on DBH growth (Jordan and Philips, 2023).

**Table 7:** Parameter estimations for the dummy variable model (Equation 8).

Model parameters	Estimation Values	Std	t value	p(r<t)
*a_1_*	4.203706	0.487757	8.618	< 2e-16 ***
*a_2_*	3.971733	0.466937	8.506	< 2e-16***
*a_3_*	3.926678	0.460988	8.518	< 2e-16***
*a_4_*	4.07862	0.477705	8.538	< 2e-16***
*a_5_*	4.119038	0.479186	8.596	< 2e-16***
*a*	1.591742	0.469297	3.392	0.000696***
*b*	0.026961	0.001668	16.159	< 2e-16***
*c*	0.856735	0.033305	25.724	< 2e-16***
*d*	-0.135598	0.00758	-17.888	< 2e-16***

‘***’ indicates significance at the p < 0.001 level.

**Table 8:** Parameter estimations for the mixed-effects model (fixed effects).

Model parameters	Estimation Values	Std.e	t value	p
a_1_	4.461787	0.9142237	4.88041	<0.0001***
a_2_	0.230677	0.056861	4.05686	0.0001***
a_3_	0.203524	0.0506867	4.015322	0.0001***
a_4_	0.190579	0.0475194	4.010551	0.0001***
a_5_	0.200793	0.0507849	3.953799	<0.0001***
a	0.213843	0.0525322	4.070705	0.0001***
b	1.461044	0.0502492	29.075945	<0.0001***
c	0.007194	0.0258796	0.277977	0.781
d	0.042946	0.0041594	10.324873	<0.0001***

‘***’ indicates significance at the p < 0.001 level.

## 4 Conclusion

A DBH estimation model was developed using airborne LiDAR to estimate individual tree DBH in large-scale Chinese fir plantations. This model integrates dummy variables and a two-level mixed-effects approach, which accounts for regional heterogeneity and the influence of tree age on the allometric growth relationship of Chinese fir. With a coefficient of determination of 0.7025, the model demonstrates strong interpretability and performs well in validation, highlighting its robust generalization ability. These findings support the potential application of airborne LiDAR in plantation inventory.

In summary, we have developed a tailored single-tree DBH model for Chinese fir plantations in southern China. The model is characterized by its simplicity, strong interpretability, and effectiveness, offering a valuable tool for improving DBH assessments in plantation inventory practices.

### 4.1 Suggestion

Establishing DBH models using airborne LiDAR and other remote sensing techniques significantly enhances the efficiency and scalability of DBH estimation. As future advancements integrate multiple remote sensing methods and improve LiDAR-derived stand structure characterization, DBH models are expected to achieve higher accuracy and progressively replace traditional field-based surveys. The Chinese fir DBH model developed in this study provides an effective approach for large-scale DBH assessment in southern China. Future research could focus on improving the representation of horizontal canopy structure in LiDAR data and integrating multi-source remote sensing data to enhance model robustness and predictive accuracy

## Data Availability

The raw data supporting the conclusions of this article will be made available by the authors, without undue reservation.
